# Metabolic differences of two constructive species in saline-alkali grassland in China

**DOI:** 10.1186/s12870-021-03401-y

**Published:** 2022-01-26

**Authors:** Qi Chen, Huansong Xie, Guanyun Wei, Xiaorui Guo, Jian Zhang, Xueyan Lu, Zhonghua Tang

**Affiliations:** 1 School of Life Sciences Nantong University, Nantong, China; 2 Key Laboratory of Plant Ecology, Northeast Forestry University, Harbin, China; 3 Northeast Agricultural University, Harbin, China

**Keywords:** Saline-alkali stress, *Puccinellia tenuiflora*, *Suaeda salsa*, Metabolomics, Tolerance

## Abstract

**Background:**

Salinization of soil is an urgent problem that restricts agroforestry production and environmental protection. Substantial accumulation of metal ions or highly alkaline soil alters plant metabolites and may even cause plant death. To explore the differences in the response strategies between *Suaeda salsa* (*S. salsa*) and *Puccinellia tenuiflora* (*P. tenuiflora*), two main constructive species that survive in saline-alkali soil, their metabolic differences were characterized.

**Result:**

Metabolomics was conducted to study the role of metabolic differences between *S. salsa* and *P. tenuiflora* under saline-alkali stress. A total of 68 significantly different metabolites were identified by GC-MS, including 9 sugars, 13 amino acids, 8 alcohols, and 34 acids. A more detailed analysis indicated that *P. tenuiflora* utilizes sugars more effectively and may be saline-alkali tolerant via sugar consumption, while *S. salsa* utilizes mainly amino acids, alcohols, and acids to resist saline-alkali stress. Measurement of phenolic compounds showed that more C6C3C6-compounds accumulated in *P. tenuiflora*, while more C6C1-compounds, phenolic compounds that can be used as signalling molecules to defend against stress, accumulated in *S. salsa.*

**Conclusions:**

Our observations suggest that *S. salsa* resists the toxicity of saline-alkali stress using aboveground organs and that *P. tenuiflora* eliminates this toxicity via roots. *S. salsa* has a stronger habitat transformation ability and can provide better habitat for other plants.

**Supplementary Information:**

The online version contains supplementary material available at 10.1186/s12870-021-03401-y.

## Background

Soil salinization is a serious environmental problem that largely restricts the production of agroforestry [1]. More than 20% of irrigated soils are affected by saline-alkali stress worldwide, and the situation is continuously deteriorating [2]. The area of salinized land is growing at a rate of 1.5 million ha per year. The increase in soluble salt in soil causes hypertonic conditions and hinders water absorption by roots. The substantial accumulation of metal ions in the cytoplasm destroys ionic equilibrium [3, 4] The change in pH leads to acid-base imbalance and damages the plant cell membrane structure [5]. These factors seriously impair land utilization. Anthropogenic disturbance and intervention further aggravate soil erosion, exacerbate land desertification, and destroy aquifer resources. Saline-alkali affected soils cause an imbalance between plants and the external environment, decrease the plant photosynthetic rate, and disrupt the normal metabolism of plants [6–8]. Saline-alkali stress reduces osmotic potential, causes ion imbalance, inhibits plant growth, and even leads to plant death [9, 10]. Salinization-induced lack of water and arid climates further affect the resistance to alkalinity and aggravate soil erosion [11].

The Hulun Buir Grassland is a famous natural pasture located in Northeast China. However, it has been reported that degradation, desertification, and salinization affect 46 million hectares in the Hulun Buir Grassland, accounting for 62.68% of the total area [12]. The increase in salinized land results in decreased production and quality of herbage. Salinization-induced damage causes vegetation degradation, induces more severe grassland salinization, and results in a low yield of pastures. Therefore, solving the soil salinization issue in the Hulun Buir Grassland is urgently needed.

There are many saline-alkali grassland communities in the Hulun Buir Grassland. These saline-alkali resistant natural plants improve the properties of soil and provide better living conditions for the other plants. The saline-alkali resistant natural plants are not only beneficial for ecological restoration but also ideal materials for studying saline-alkali stress. *Suaeda salsa* (*S. salsa*) and *Puccinellia tenuiflora* (*P. tenuiflora*) are two very important saline-alkali tolerant plants and community-building species in the Hulun Buir Grassland. *S. salsa* is recognized as the first-line warrior to defend against saline-alkali stress, it exhibits high salt tolerance during germination, growth, and reproduction [13–15]. The Na^+^ content even reached 60 mg/g and 45 mg/g in *S. salsa* under 300 mM NaCl treatment and 200 mM NaHCO_3_ treatment, respectively [15]. *P. tenuiflora* generally grows in degraded grasslands or salinized soils, it has strong saline-alkali resistance and is known as a “pioneer in saline-alkali grass” [16, 17]. *P. tenuiflora* can grow well in soil with a pH value higher than 10 and a salt content greater than 5% [18]. Experimental results have shown that the Na^+^ content can reach 50 mg/g and 40 mg/g in *P. tenuiflora* under 100 mM NaCl treatment and 50 mM NaHCO_3_ treatment, respectively [19]. Considering that *S. salsa* and *P. tenuiflora* have strong adaptability to saline-alkali stress and can effectively improve the surrounding environment, it is important to explore the response mechanisms of *S. salsa* and *P. tenuiflora* under saline-alkali stress. In the current study, we collected *S*. *salsa* and *P. tenuiflora* from their communities and determined their metabolic changes, aiming to reveal the differences in their saline-alkali responses. Our study may help to seek a way to improve vegetation restoration, increase crop yield, and encourage the sustainable development of agriculture.

## Result

### Overview of *S. salsa* and *P. tenuiflora* communities

**Fig. 1:**
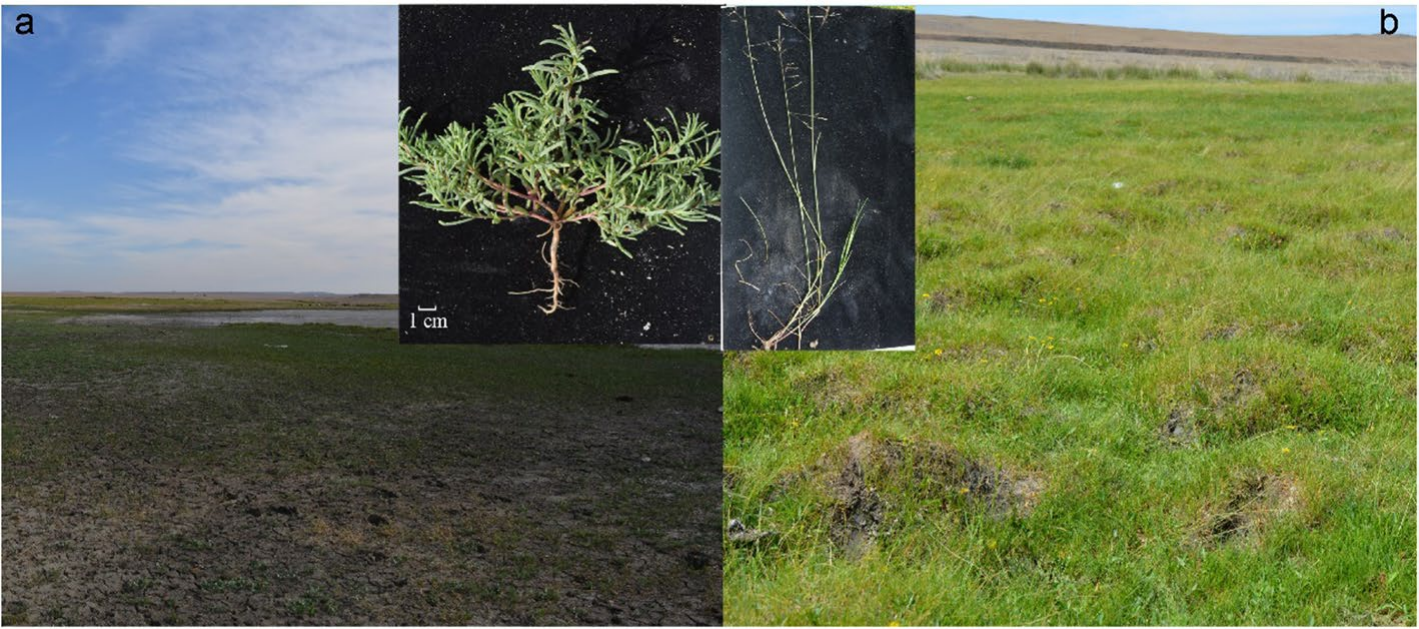
The appearance of *Suaeda salsa* community and *Puccinellia tenuiflora* community. (a) *S. salsa* community and *S. salsa*; (b) *P. tenuiflora* community and *P. tenuiflora*

The *P. tenuiflora* community shows more species diversity and land cover than the *S. salsa* community (Fig. 1). Soils around the *S. salsa* community present “leucophylline” and harden into a lump, indicating that *S. salsa* faces more serious saline-alkali stress. According to the soil alkalization classification standard (SSC), both the rhizosphere soils of *S. salsa* and *P. tenuiflora* belong to salinization soils (Table 1). The alkalization of *S. salsa* rhizosphere soil was significantly higher than that of *P. tenuiflora* rhizosphere soil and was classified as severe salinization (pH > 9.0) (Table 1).

**Table 1:** The soil indicator around *S. salsa* and *P. tenuiflora* (mg/g)

	pH	Na^+^(%)	K^+^(%)	Na^+^/K^+^	CO_3_^2−^(%)	HCO_3_^−^(%)	Cl^−^(%)	SO_4_^2−^(%)
S1	9.74 ± 0.14**	0.35 ± 0.07**	0.01 ± 0	35 ± 2**	0.018 ± 0.003	0.037 ± 0.009**	0.090 ± .014*	0.20 ± 0.021
S2	8.53 ± 0.25	0.15 ± 0.005	0.006 ± 0	25 ± 3	0.015 ± 0.001	0.005 ± 0.001	0.037 ± 0.009	0.09 ± .011

S1: the soil around *S. salsa*, S2: the soil around *P. tenuiflora*. *, *p* < 0.05; **, *p* < 0.01

### The responses of primary metabolites to saline-alkali stress

**Fig. 2:**
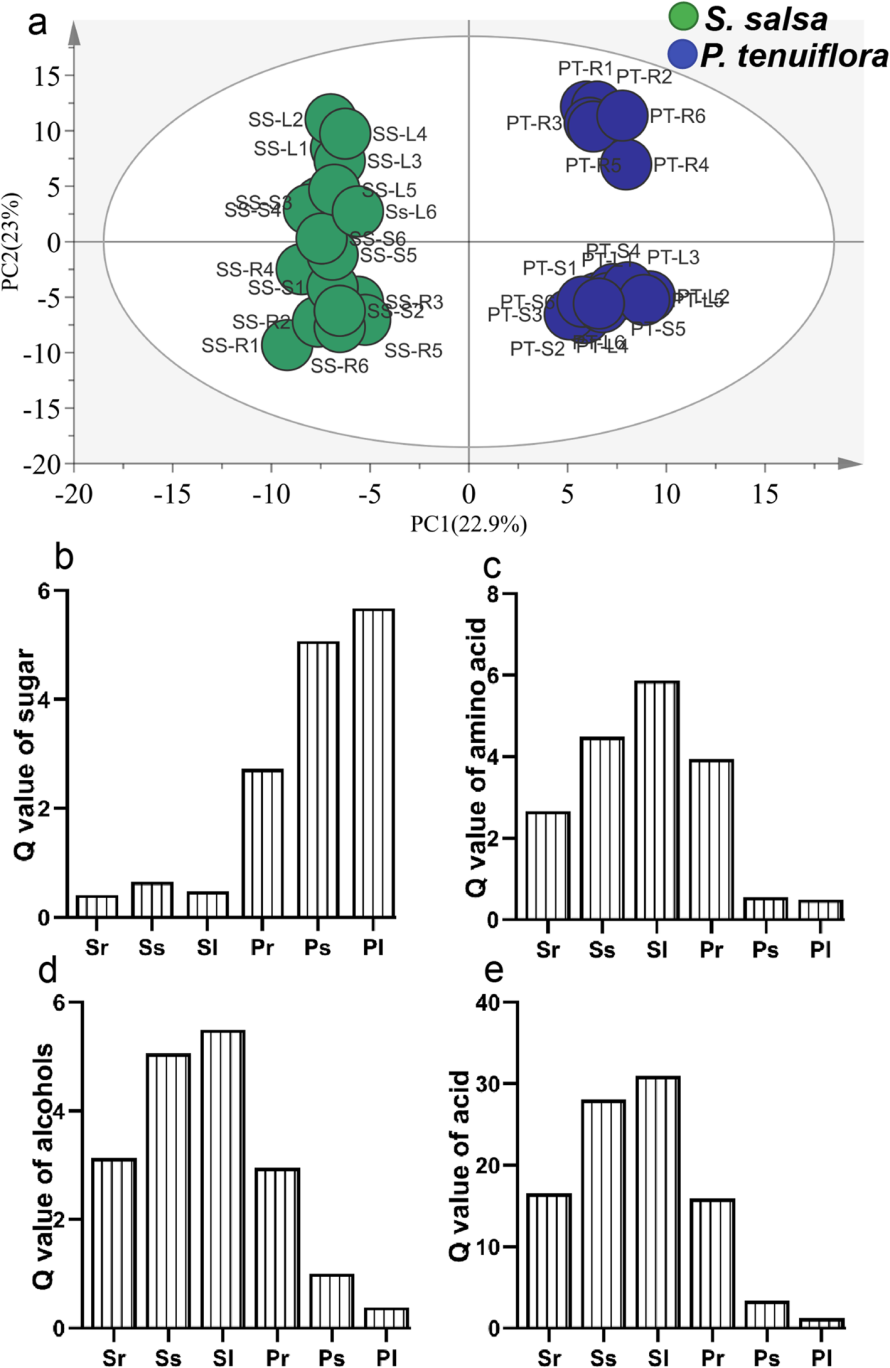
The OPLS-DA score plot of primary metabolites and the Q value of them significantly metabolites between *S. salsa* and *P. tenuiflora*. (a) The OPLS-DA score plot of primary metabolites; (b) Q value of sugar; (c) Q value of amino acid; (d) Q value of alcohol; (e) Q value of acids. Sr: the root of *S. salsa*. Ss: the stem of *S. salsa*. Sl: the leaf of *S. salsa*. Pr: the root of *P. tenuiflora*. Ps: the stem of *P. tenuiflora*. Pl: the leaf of *P. tenuiflora*

To explore the differences in saline-alkali resistance between *S. salsa* and *P. tenuiflora*, GC-MS was used to detect the amounts of metabolites related to the response to saline-alkali stress. *S. salsa* and *P. tenuiflora* were clearly separated by means of PC1 (22.9%) and PC2 (23.0%) with OPLS-DA, a supervised method that can classify observations into the group with the largest predicted indicator variable (Fig. 2. a). A total of 68 significantly different metabolites between *S. salsa* and *P. tenuiflora* were obtained according to their variable importance in the projection (VIP, VIP > 1) and *p*-values (*p* < 0.05). These significantly different metabolites could be classified into 9 sugars, 13 amino acids, 8 alcohols, 34 acids, and 4 other compounds (Table S1). The calculation of the principal component Q value showed that sugars were obviously accumulated in *P. tenuiflora* (Fig. 2b), while other primary metabolites, i.e., amino acids, alcohols, and acids, were all significantly higher in *S. salsa* than in *P. tenuiflora* (Fig. 2c-e). The distributions of these primary metabolites were also different in different parts (root, stem, and leaf) of plants. For instance, the root of *P. tenuiflora* had the lowest Q value of sugars, while the leaf of *P. tenuiflora* had the highest Q values of sugars (Fig. 2b). In contrast, the root of *P. tenuiflora* had the highest Q values of amino acids, alcohols, and acids, while the leaf of *P. tenuiflora* had the lowest Q values (Fig. 2c-e).

**Fig. 3:**
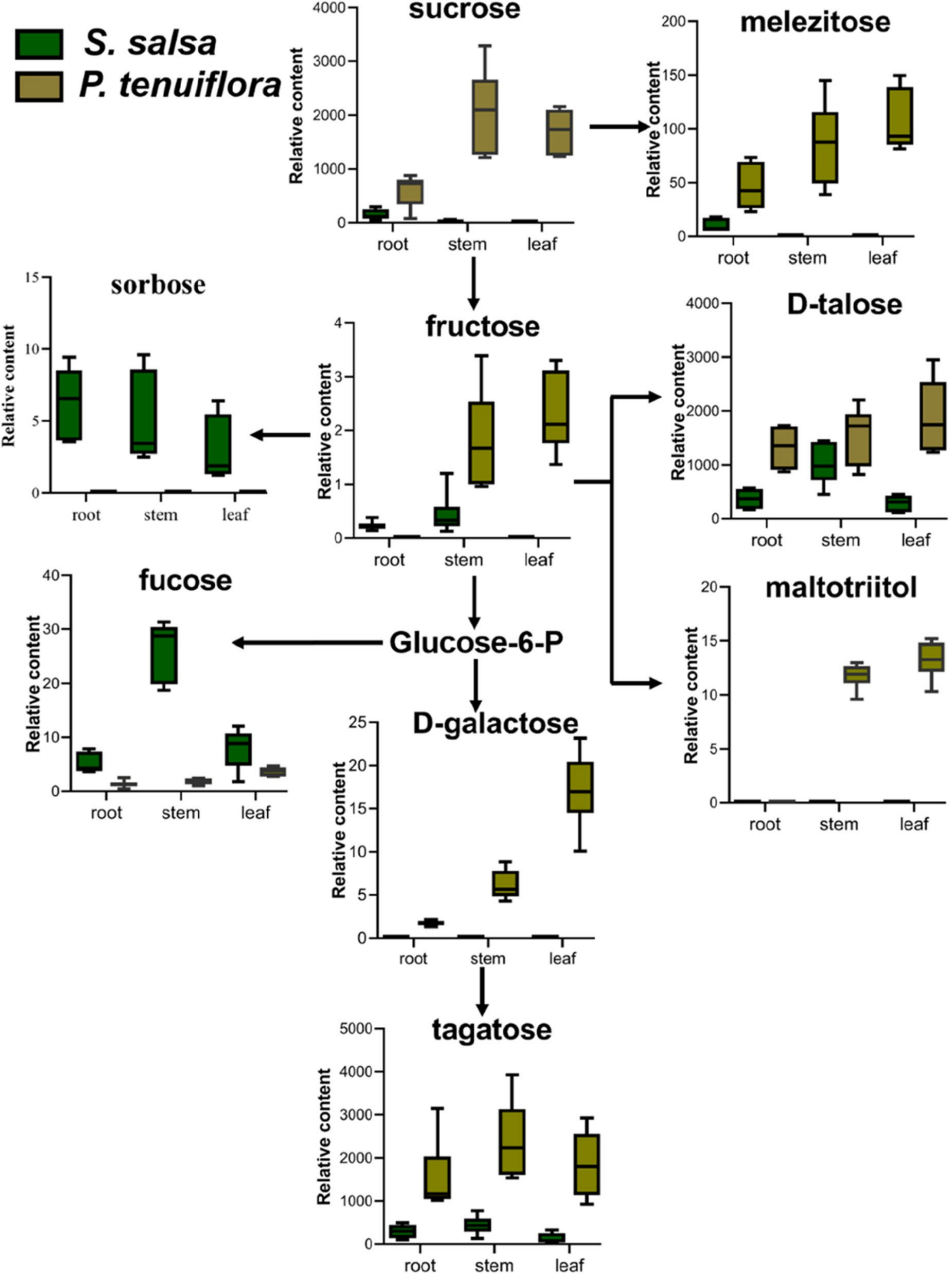
The metabolic network of significantly sugars between *S. salsa* and *P. tenuiflora*

The distributions of these primary metabolisms were further analysed. The sugars showed that among 9 significantly different sugars, only two kinds of 6-carbon sugars, sorbose and fucose, were enriched in *S. salsa* (Fig. 3). The other 7 kinds of sugars were all highly accumulated in *P. tenuiflora*, including 6-carbon sugars tagatose, D-talose, fructose, and D-galactose, 12-carbon sugars sucrose and maltotriitol, and 18-carbon sugar melezitose (Fig. 3). These sugars not only provide energy in plants but also play a key role in resisting saline-alkali stress.

**Fig. 4:**
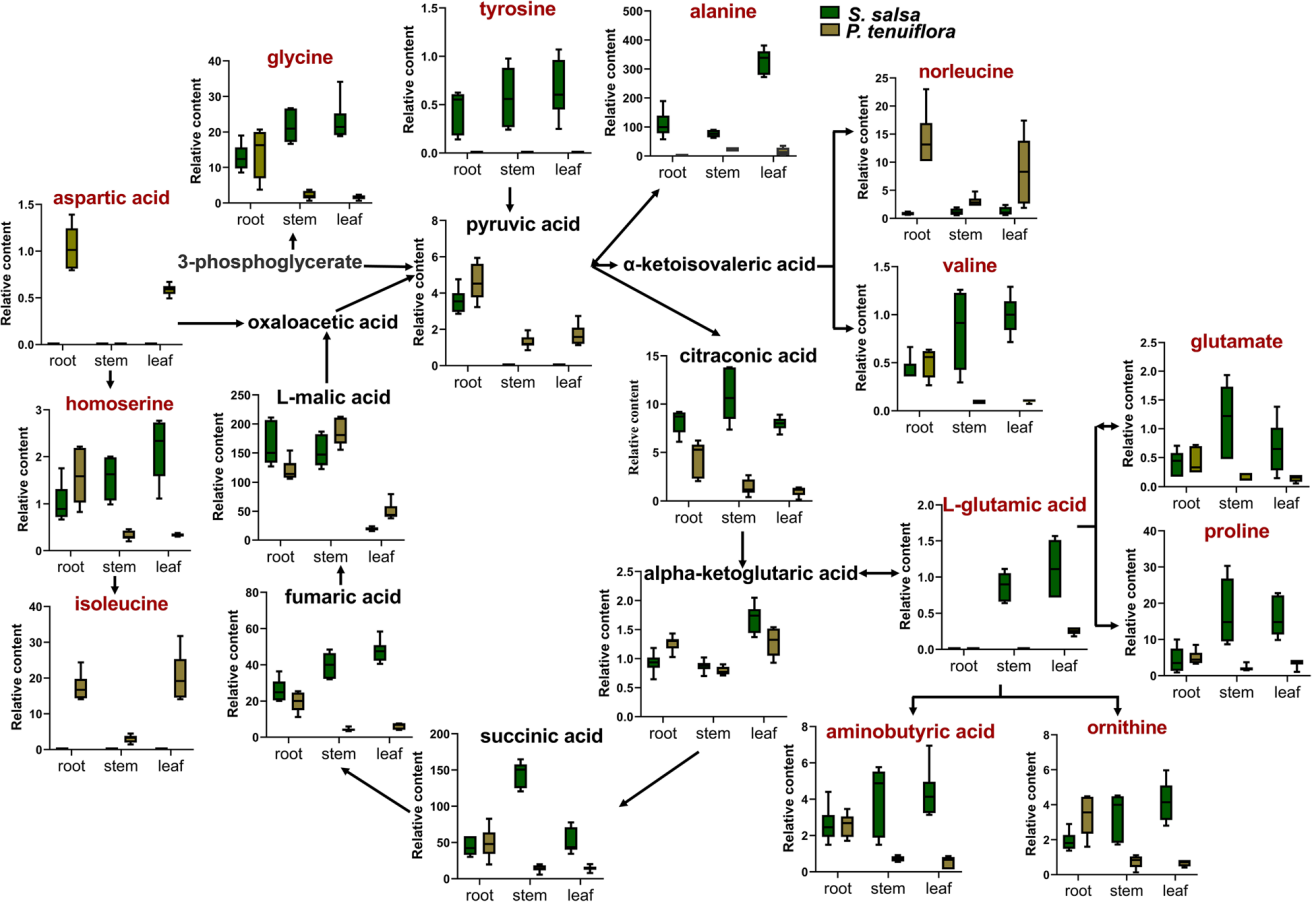
The metabolic network of amino acids between *S. salsa* and *P. tenuiflora*. The significantly different metabolites were marked red

In contrast to sugars, detailed analyses of differentially expressed amino acids showed that most kinds of amino acids were remarkably accumulated in *S. salsa* (Fig. 4). Only isoleucine, norleucine, and aspartic acid accumulated at higher levels in *P. tenuiflora*, with isoleucine largely accumulating in the leaf and norleucine and aspartic acid largely accumulating in the root (Fig. 4). Other amino acids were discovered to be highly accumulated in *S. salsa*, especially in the aboveground part of *S. salsa* (Fig. 4). These amino acids play an important role in osmoregulation under saline-alkali stress.

**Fig. 5:**
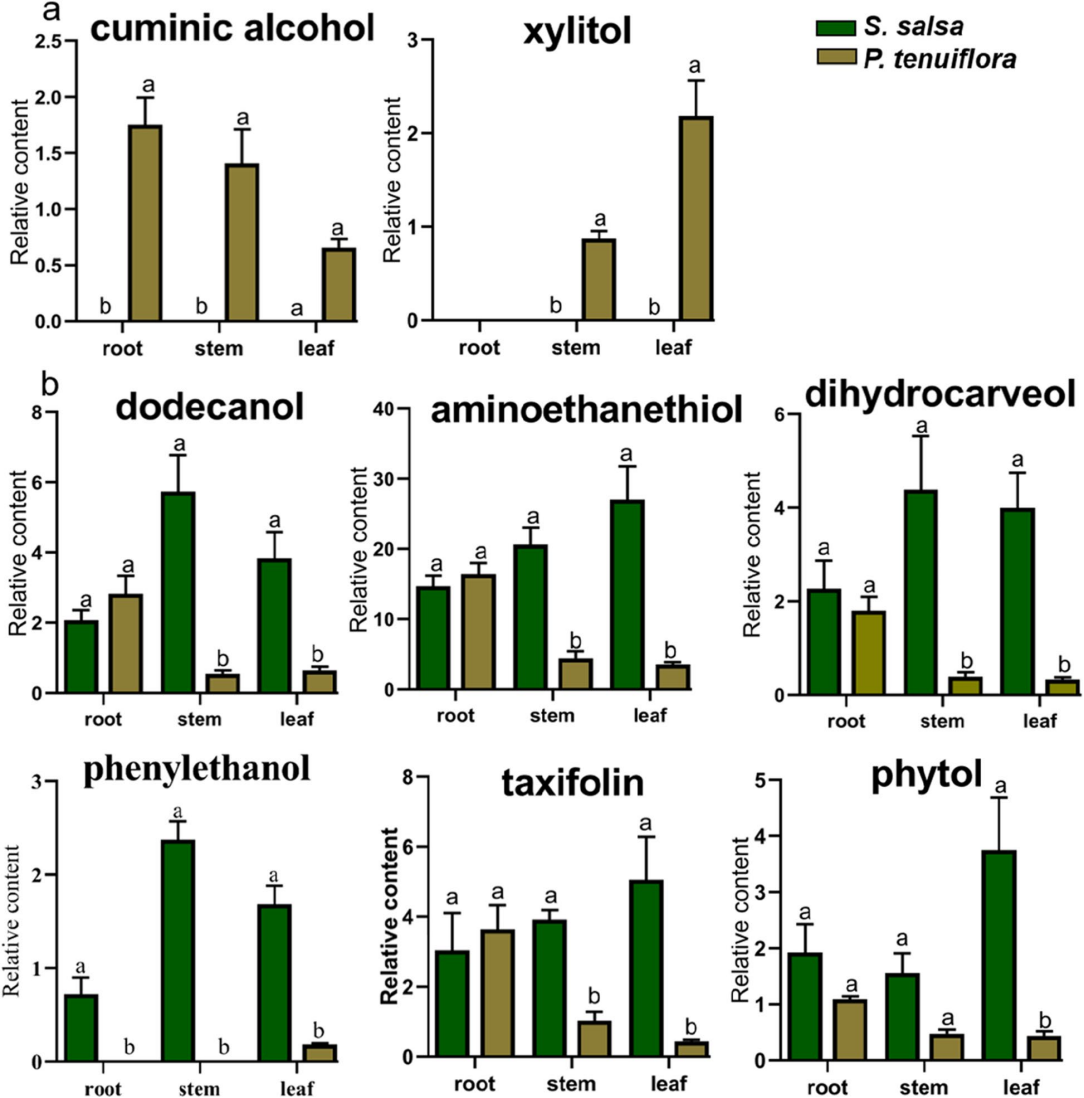
The significantly different alcohols between *S. salsa* and *P. tenuiflora*. (a) The significantly different alcohols mainly accumulated in *P. tenuiflora*; (b) The significantly different alcohols mainly accumulated in *S. salsa*. The relative contents of significantly different alcohols are summarized from 6 biological replicates and presented as the mean ± standard error of 6 biological replicates. Different letters indicate significant differences among treatments (*p* < 0.05)

Similar to amino acids, the majority of differentially expressed alcohols showed high abundances in *S. salsa* (Fig. 5). These alcohols mainly accumulated in the aboveground part of *S. salsa*. Two other alcohols, cuminic alcohol and xylitol, were found to accumulate in *P. tenuiflora* (Fig. 5). Cuminic alcohol was enriched in the root of *P. tenuiflora,* while xylitol was enriched in the leaf.

Acids accounted for a large proportion of significantly different metabolites (Table 2). Differentially expressed acids were artificially grouped into phenolic compounds, organic acids, and volatile compounds. Detailed investigation of the organ-specific expression of acids showed that acids accumulated largely in the aboveground part of *S. salsa* and the root of *P. tenuiflora* (Table 2).

**Table 2:** Significantly different acids in root, stem, and left of *S. salsa* and *P. tenuiflora*

Acids		*S. salsa*	SSC (%)		*P. tenuiflora*	SSC (%)	
			0.71			0.52	
		root	stem	leaf	root	stem	leaf
phenolic compounds	gallic acid	28 ± 3^a^	54 ± 6^a^	77 ± 12.26^a^	23 ± 7^a^	8.26 ± 3^b^	11 ± 3^b^
	protocatechuic acid	5.2 ± 2 ^a^	4.86 ± 2 ^a^	8.41 ± 2 ^a^	5.12 ± 1 ^a^	0.95 ± 0 ^b^	0.77 ± 0 ^b^
	catechol	0.42 ± 0 ^a^	1.87 ± 1 ^a^	1.42 ± 1 ^a^	0 ^b^	0.29 ± 0 ^b^	0.24 ± 0 ^b^
	epigallocatechin	1.30 ± 0 ^a^	2.34 ± 0 ^a^	4.92 ± 1 ^a^	1.01 ± 0 ^a^	0.22 ± 0 ^b^	0 ^b^
	vanillic acid	2.01 ± 1 ^a^	4.47 ± 2 ^a^	7.11 ± 2 ^a^	1.73 ± 0 ^a^	0.48 ± 0 ^b^	0.48 ± 0 ^b^
	vinylphenol	5.88 ± 1 ^a^	7.69 ± 1 ^a^	7.55 ± 1 ^a^	6.15 ± 2 ^a^	1.9 ± 1 ^b^	1.77 ± 1 ^b^
	guaiacol	0.96 ± 0 ^a^	1.78 ± 0 ^a^	2.23 ± 1 ^a^	1.03 ± 0 ^a^	0.24 ± 0 ^b^	0.22 ± 0 ^b^
organic acids	citraconic acid	8.21 ± 1 ^a^	11 ± 3^a^	7.97 ± 1 ^a^	4.49 ± 2 ^b^	1.43 ± 1 ^b^	1 ± 0 ^b^
	malonic acid	35 ± 11 ^a^	44 ± 17 ^a^	76 ± 23 ^a^	31 ± 8 ^a^	15 ± 5 ^b^	16 ± 2 ^b^
	succinic acid	44 ± 13 ^a^	145 ± 18 ^a^	52 ± 17 ^a^	48 ± 18 ^a^	16 ± 3 ^b^	15 ± 4 ^b^
	tartaric acid	1.02 ± 0 ^a^	2.43 ± 1 ^a^	1.75 ± 1 ^a^	0.98 ± 0 ^a^	0 ^b^	0.33 ± 0 ^b^
	itaconic acid	21 ± 5 ^a^	40 ± 9 ^a^	28 ± 9 ^a^	19 ± 7 ^a^	3.38 ± 1 ^b^	4.44 ± 2 ^b^
	pelargonic acid	0.52 ± 0 ^a^	0.88 ± 0 ^a^	1.91 ± 1 ^a^	0.91 ± 0 ^a^	0.31 ± 0 ^a^	0.19 ± 0 ^b^
	glycolic acid	17 ± 6 ^a^	41 ± 11 ^a^	56 ± 8 ^a^	20 ± 8 ^a^	6.31 ± 2 ^b^	10 ± 2 ^b^
	3-methylglutaric acid	1.46 ± 1 ^a^	0 ^a^	1 ± 0.58 ^a^	0 ^b^	0 ^a^	0 ^b^
	aminooxyacetic acid	12 ± 4 ^a^	13 ± 4 ^a^	18 ± 7 ^a^	8.44 ± 4 ^a^	0.79 ± 0 ^b^	0.63 ± 0 ^b^
	oxalic acid	2.58 ± 1 ^a^	3.63 ± 1 ^a^	4.01 ± 15 ^a^	3 ± 0^a^	0.81 ± 1 ^b^	1.15 ± 0 ^b^
	L-gulonic acid	2.5 ± 1^b^	3.84 ± 1 ^a^	2.01 ± 0 ^b^	13 ± 5 ^a^	10 ± 3 ^a^	10 ± 2 ^a^
	cumic acid	4.15 ± 1 ^a^	6.76 ± 2 ^a^	9.27 ± 3 ^a^	5.43 ± 2 ^a^	1.43 ± 1 ^b^	0.63 ± 0 ^b^
	palmitic acid	295 ± 86^a^	401 ± 42^a^	577 ± 140^a^	508 ± 215^a^	100 ± 31^b^	77 ± 18^b^
volatile compounds	methylfumarate	0.94 ± 0 ^a^	1.83 ± 0 ^a^	1.46 ± 0 ^a^	0.71 ± 0 ^a^	0.16 ± 0 ^b^	0.16 ± 0 ^b^
	hydroxybutyrate	0 ^b^	0 ^b^	0.32 ± 0 ^a^	0.35 ± 0 ^a^	0.16 ± 0 ^a^	0.31 ± 0 ^a^
	gluconic lactone	2.89 ± 1 ^a^	2.46 ± 1 ^a^	0.98 ± 0 ^a^	0 ^b^	0 ^b^	0 ^b^
	methyl hexadecanoate	0.33 ± 0 ^a^	0.52 ± 0 ^a^	0.92 ± 0 ^a^	0 ^b^	0 ^b^	0.11 ± 0 ^b^
	dioctyl phthalate	9.13 ± 3 ^a^	13 ± 3 ^a^	20 ± 6 ^a^	8.92 ± 2 ^a^	1.39 ± 1 ^b^	1.24 ± 0 ^b^
	methyl heptadecanoate	3.76 ± 1 ^a^	8.3 ± 1 ^a^	4.16 ± 1 ^a^	1.87 ± 1 ^b^	0.69 ± 0 ^b^	0.44 ± 0 ^b^
	nonanoic acid methyl ester	79 ± 18 ^a^	105 ± 23^a^	149 ± 29^a^	114 ± 33 ^a^	23 ± 7^b^	19 ± 5 ^b^
	methyl octanoate	44 ± 6 ^a^	59 ± 13 ^a^	92 ± 17 ^a^	62 ± 19 ^a^	14 ± 3^b^	10 ± 3 ^b^
	L-gulonolactone	0 ^a^	0 ^b^	0 ^b^	0 ^a^	1.61 ± 0 ^a^	8.46 ± 4 ^a^
	phenylacetic acid	0 ^b^	0.64 ± 0 ^a^	0.64 ± 0 ^a^	0.33 ± 0 ^a^	0 ^b^	0 ^b^
	hydroxymandelic acid	11 ± 2 ^a^	8.9 ± 1 ^a^	14 ± 4 ^a^	12 ± 5 ^a^	3.24 ± 1 ^a^	2.1 ± 1 ^b^
	5-hydroxyindole-2-carboxylic acid	0.73 ± 0 ^a^	1.57 ± 1 ^a^	1.89 ± 1 ^a^	0 ^a^	0.22 ± 0 ^b^	0.36 ± 0 ^b^
	5-hydroxyindole-3-acetic acid	1.41 ± 1 ^a^	3.17 ± 1 ^a^	2.39 ± 0 ^a^	1.37 ± 0 ^a^	0.27 ± 0 ^b^	0 ^b^

SSC: Soluble salt content. The relative contents of acids are summarized from 6 biological replicates and presented as the mean ± standard error of 6 biological replicates. Different letters indicate significant differences of the same tissues between *S. salsa* and *P. tenuiflora* (p < 0.05)

### The responses of phenolic compounds to saline-alkali stress

**Fig. 6:**
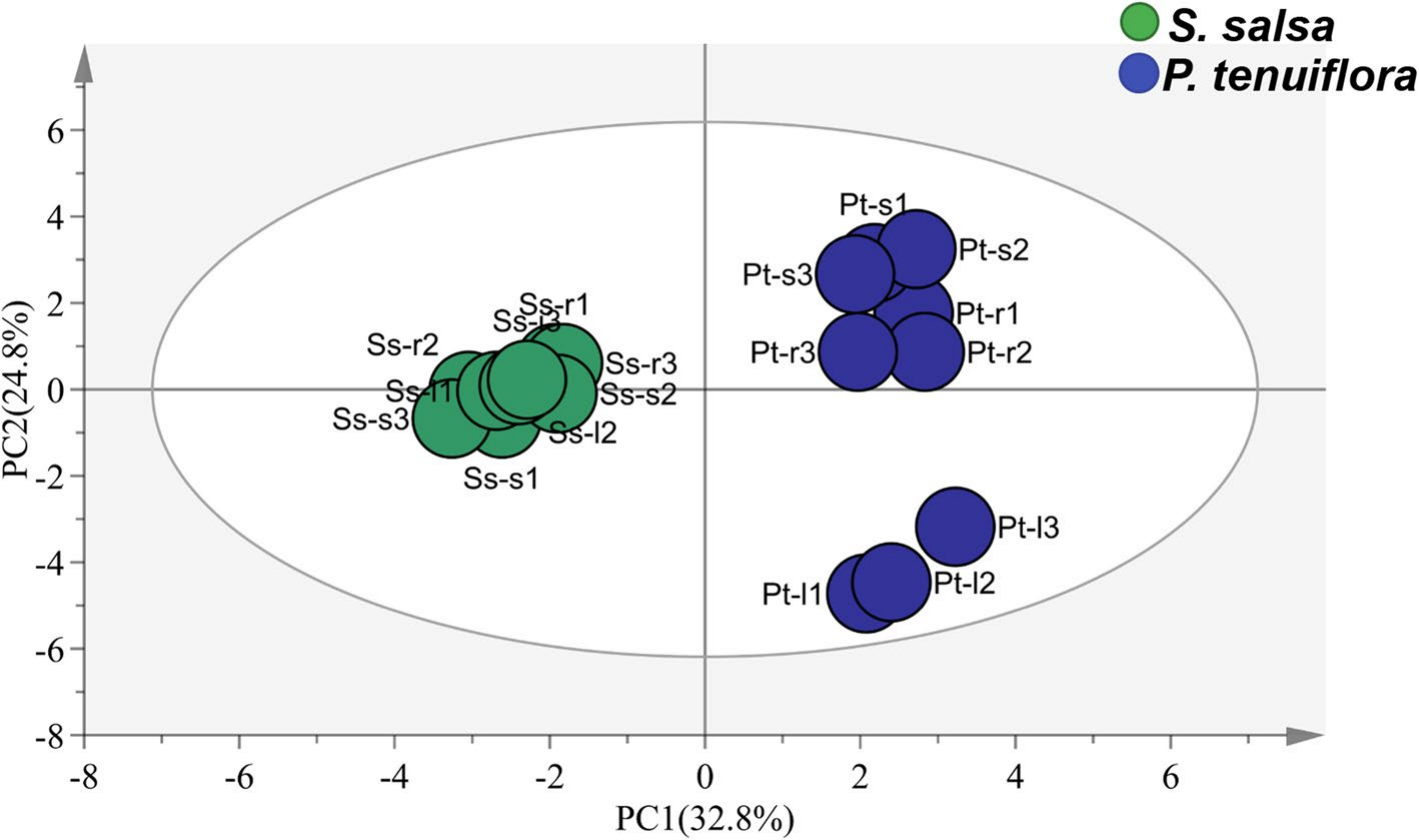
The OPLS-DA score plot of phenolic compounds

**Fig. 7:**
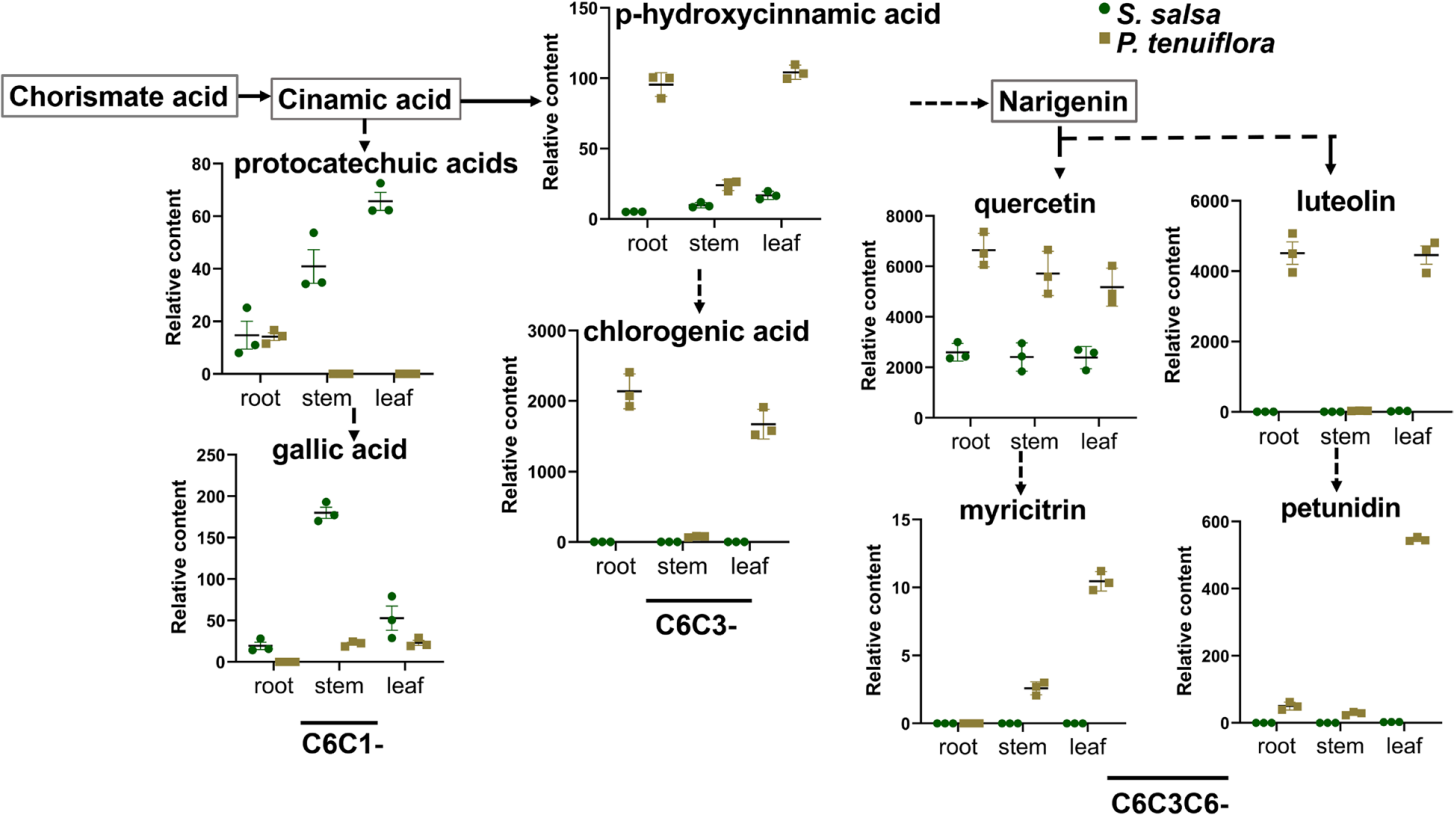
Visualization of the difference of significantly phenolic compounds on a biochemical pathway map

Phenolic compounds from phenylalanine metabolism are important for plant development and defence and play an essential role in saline-alkali stress. To explore the involvement of phenolic compounds in saline-alkali stress, HPLC-qTOF-MS was performed, and the accumulation of a total of 34 phenolic compounds were measured. Twenty phenolic compounds were found to accumulate in *S. salsa* and *P. tenuiflora*, and 8 phenolic compounds were identified to be significantly differentially expressed (VIP > 1 and *p* < 0.05) by OPLS-DA (Fig. 6). These 8 significantly different phenolic compounds could be divided into 2 C6C1-compounds (protocatechuic acid and gallic acid), 2 C6C3-compounds (chlorogenic acid, *p*-hydroxycinnamic acid), and 4 C6C3C6-compounds (luteolin, quercetin, myricitrin, and petunidin) according to their carbon skeletons (Fig. 7). C6C1-compounds were found to notably accumulate in *S. salsa*, especially the aboveground part of *S. salsa,* while C6C3-compounds and C6C3C6-compounds were identified to be mainly enriched in *P. tenuiflora* (Fig. 7).

## Discussion

Saline-alkali tolerant plants show excellent potential for preventing soil salinization, improving the ecological environment, and providing live conditions for other plants that have lower tolerance to saline-alkali stress [2]. Emerging studies have focused on the biological responses of saline-alkali tolerant plants, aiming to decipher saline-alkali tolerant mechanisms. For example, *Chenopodium quinoa* Wild. has been used to investigate the genotype-dependent variability in salinity responses from morphological, physiological, cellular, and molecular aspects [20]. However, these plant tolerance or defence studies are conducted mainly by indoor control [16, 21, 22]. The application of indoor control cannot fully reflect the natural responses of plants to saline-alkali stress, as plants that survive in saline-alkali soil usually undergo long-term adaptation and evolution with habitat. In the current study, we measured the expression of metabolites in *S*. *salsa* and *P. tenuiflora* that survive in saline-alkali soil using GC-MS and LC-qTOF-MS and demonstrated diverse metabolites with varied intensities in *S*. *salsa* and *P. tenuiflora*.

Many sugars have been identified as regulatory components in the control of glycolytic flux in a variety of stress survival strategies [23]. These sugars not only act as readily available energy sources for plant growth under stress but also function as osmoprotectants to maintain osmotic balance and stabilize macromolecules [24]. Soluble sugars provide an adaptive buffer for plants under saline-alkali stress and play an important role in regulating osmotic pressure [25, 26]. In our studies, many soluble sugars, including sorbose, fucose, and D-talose, were highly expressed. They have the ability to balance osmotic pressure and protect the biological structures of plants from desiccation damage [27, 28]. Notably, many metabolites in glycolysis/gluconeogenesis pathways were found to be significantly accumulated in *P. tenuiflora*, indicating that the production of downstream products through metabolic flux from these pathways is essential for saline-alkali tolerance. Therefore, it is likely that *P. tenuiflora* can regulate central metabolism by effectively utilizing carbon, accumulating carbon assimilation production, and providing more material and energy to promote tolerance against saline-alkali stress.

Nitrogen metabolism has been reported to be strongly interconnected with carbon metabolism [29]. A sufficient carbon skeleton source and energy supply are important for the assimilation of nitrogen and the synthesis of amino acids [30]. Plants respond to high salinity by limiting protein synthesis, promoting protein degradation, and changing their amino acid compositions [31, 32]. Here, significant enrichment of amino acids was discovered in *S. salsa*. Therefore, we speculate that *S. salsa* uses a different saline-alkali response strategy from that of *P. tenuiflora.* In *P. tenuiflora*, a large portion of carbon influx to sugars occurred, while only the amino acids isoleucine, norleucine, and aspartic acid were highly accumulated (Fig. 3a). Isoleucine and norleucine can improve salt resistance and maintain metabolic and osmotic homeostasis under stress [24]. Aspartic acid can act as an immediate donor of amino groups for the synthesis of other amino acids [30]. Many other amino acids, including glutamine, proline, alanine, tyrosine, ornithine, and 3-hydroxynorvaline, were identified to be significantly accumulated in *S. salsa*. Glutamine has an elevated nitrogen-to-carbon ratio and can use limited carbon skeletons to respond to environmental stresses [33, 34]. Proline is generally considered an osmotic regulator and an active oxygen scavenger in response to high salinity [35]. Similar to molecular chaperones, proline can form a protective film [36]. Proline is produced mainly by the glutamate synthesis pathway and ornithine synthesis pathway [36]. Activation of the ornithine synthesis pathway also plays a vital role in improving plant salt tolerance. Notably, aminobutyric acid, which is involved in various stress response and defence mechanisms, also accumulates considerably in *S. salsa*. Aminobutyric acid can maintain carbon and nitrogen balance, protect plants from oxidative stress, and regulate the pH value of the cytoplasm. These differentially regulated amino acids help *S. salsa* survive under saline-alkali stress.

Alcohols help to reserve available water in plants and thus are considered essential osmotic regulators. In our current study, accumulated alcohols in the aboveground part of *S. salsa* were discovered (Fig. 5). These alcohols benefit the maintenance of osmotic pressure balance in the cytoplasm and contribute to the regulation of water loss [37]. In addition, alcohols work as natural scavengers of salinity-induced reactive oxygen species and protect biomolecules against oxidative damage [38].

The roles of soluble sugars, alcohols, and amino acids in resisting saline-alkali stress have been well acknowledged. Our current study revealed that a large proportion of differentially expressed metabolites were acids, implying the potential involvement of acids in plant protection. Acids can enhance plant stress resistance and stabilize intracellular pH [39]. We found that many acids accumulated in the aboveground part of *S. salsa*. These acids may help to maintain ionic balance by neutralizing alkali and excess toxic ions. They can also affect the fluidity and hydrophobicity of the cell membrane, which is crucial for cell membrane activity maintenance and saline-alkali stress defence [40]. Notably, some acids, such as nonanoic acid methyl ester, methyl hexadecanoate, and phenylacetic acid, have obvious flavours and are volatile. The secretion of these acids may affect the surrounding environment and influence soil composition (Table 2). Volatile substances are also communication factors that contribute to plant defence and reproduction [41]. Therefore, these volatile substances may improve soil properties via signal transmission and communication. The pioneering role of *S. salsa* may be partially attributed to the successful secretion of these allelopathic compounds under saline-alkali stress.

Our GC-MS results showed that gallic acid, vanillic acid, protocatechuic acid, and catechol, acids subordinated to phenolic compounds, were obviously accumulated. These compounds are secondary metabolites that originate from phenylalanine metabolism [42–44]. Gallic acid and protocatechuic acid are the precursors of tannins, which can affect plant thickness and reduce water evaporation [20]. Moreover, a larger number of other phenolic compounds were investigated. Bioactive phenolic compounds are important biofactories of plants under stress [45, 46]. These compounds are divided mainly into benzoic acid derivatives with C6C1 carbon skeletons (C6C1-compounds), hydroxycinnamic acid derivatives with C6C3 carbon skeletons (C6C3-compounds), and flavonoids with C6C3C6 carbon skeletons (C6C3C6-compounds). Our results demonstrated the enrichment of C6C1-compounds in *S. salsa* as well as the enrichment of C6C3- and C6C3C6-compounds in *P. tenuiflora* (Fig. 7). C6C1-compounds, usually induced by biotic elicitors, are signalling molecules that defend against stress. C6C3C6-compounds are flavonoids that can directly enhance the chemical defence of plants and help plants adapt to their environments [47]. Significant accumulation of phenolic compounds in *S. salsa* and *P. tenuiflora* may thus benefit their saline-alkali tolerance.

## Conclusions

Our results showed that *S. salsa* resists the toxicity of saline-alkali stress using aboveground organs and that *P. tenuiflora* eliminates this toxicity via roots. *S. salsa* has a stronger habitat transformation ability and is more tolerant to saline-alkali conditions than *P. tenuiflora*. The analyses of different metabolites of *S. salsa* and *P. tenuiflora* provide an important theoretical basis for understanding the mechanisms of saline-alkali tolerance and may help to deepen the knowledge of plant metabolism regulation under stress.

## Materials and methods

### Materials collected

*S*. *salsa* and *P. tenuiflora* were selected from *S*. *salsa* and *P. tenuiflora* communities of the Hulun Buir Grassland in China (115°31′00″-121°34′30″, 47°20′00″-50°50′30″). Xie Huansong identified *S*. *salsa* and *P. tenuiflora*. Samples were collected from three different plots with similar transitional communities from *S. salsa* to *P. tenuiflora*. Distances between sample plots were greater than 500 km. Three plants were selected from each community with 6 repeats and stored in liquid nitrogen. Soil samples derived from the habitats of *S. salsa* and *P. tenuiflora* were collected along the vertical length of 20 cm depth for characterizing salinization with 3–6 repeats.

### The detection of soil salinity and alkalinity

Soil samples were dried at room temperature for 2 weeks, pulverized, and sieved through a 2 mm mesh sieve. Saturation paste extract was prepared for soil detection. Soil samples were digested with the HF-HClO_4_-HNO_3_ method. The contents were determined by flame photometry (410, Corning, Halstead, England), colorimetry (double beam spectrophotometer, UV-140-02, Shimadzu), and titration method (carbonates and bicarbonates, and chlorides). The pH of the soil was measured with a glass electrode pH meter (pHM-2000, Eyela, Rikakikai Co., Tokyo, Japan) during saturation paste titration.

### GC-MS analysis

GC-MS was performed as previously described [23]. Sixty milligram samples were mixed with 360 μL cold methanol and 40 μL internal standards. Samples were homogenized (Tissuelyser-192, Shanghai, China), ultrasonicated for 30 min, mixed with 200 μL chloroform and 400 μL water, and centrifuged at 10,000×g for 10 min at 4 °C. Finally, 400 μL supernatant was transferred to a glass sampling vial for vacuum drying at room temperature. The residue was derivatized using a two-step procedure. First, 80 μL methoxyamine (15 mg/mL in pyridine) was added to the vial and maintained at 37 °C for 90 min, followed by addition of 80 μL BSTFA (1% TMCS) and 20 μL *n*-hexane at 70 °C for 60 min. After derivatization, 1 μL solution was injected into the Agilent 7890A-5975C GC-MS system (Agilent Corporation, USA) with a split ratio of 30 to 1. Separation was carried out on a non-polar DB-5 capillary column (30 m × 250 μm I.D., J&W Scientific, Folsom, CA) with high purity helium as the carrier gas at a constant flow rate of 1.0 mL/min. The temperatures of the injector and ion source were set to 260 °C and 230 °C, respectively. Electron impact ionization (− 70 eV) in full scan mode (m/z 30–600) was used, with an acquisition rate of 20 spectra/s in the MS setting. QC samples were prepared by mixing aliquots of tissue samples to be pooled samples and analysed using the same method as the analytical samples.

Acquired MS data were analysed by Chroma TOF software (v 4.34, LECO, St. Joseph, MI). Briefly, after alignment with the Statistic Compare component, the CSV file was obtained with three-dimensional datasets, including sample information, retention time, and peak intensities. The internal standard was used for data quality control (reproducibility). Internal standards and any known pseudo-positive peaks, such as peaks caused by noise, column bleed, and the BSTFA derivatization procedure, were removed from the dataset. The dataset was normalized using the sum intensity of the peaks in each sample.

### Phenolic compound detection

Phenolic compound detection was performed as previously described [42]. After treatment with liquid nitrogen, a 1.0 g pulverized sample was dissolved in 20 mL methanol for extraction and ultrasonicated at low frequency for 40 min. The simple solution was centrifuged for 10 min at 8000 rpm. The analysis was performed by a Waters ACQUITY UPLC system (Waters, Japan) coupled to a quadrupole time-of-flight (qTOF) mass spectrometer (XEVO G2 QTOF, Waters). The chromatographic conditions were as follows: A%: 0.05% formic acid-water; B%: 0.05% formic acid-acetonitrile; m/z: 120–1200; positive scan mode; and chromatographic columns: ACQUIT UPLC-BEH C18 Column (1.7 mm, 2.1 mm, × 50 mm). Leu-Enkephalin was used as the internal standard.

### Statistical analysis

Datasets obtained from GC-MS and LC-qTOF-MS were imported into the SIMCA-P14.1 software package (Umetrics, Umeå, Sweden). After mean centring and unit variance scaling, orthogonal partial least-squares discrimination analysis (OPLS-DA) was carried out to visualize metabolic alterations among experimental groups. Differentially expressed compounds were selected by comparing compounds in different groups using the multivariate statistical method. Metabolites with both multivariate and univariate statistical significance (VIP > 1.0 and *p* < 0.05) were screened. Default 7-round cross-validation was applied, with 1/7 of the samples being excluded from the mathematical model in each round to avoid overfitting.

Data were log^2-^transformed to improve normality, and min-max normalization was performed. Data were subjected to hierarchical clustering analysis by R software to study the variations of *S. salsa* and *P. tenuiflora*. Significantly different metabolites were screened by SIMICA14.1. The principal component Q score was calculated using SPSS version 21.0 software (Chicago, IL, USA). Histograms, and pathway maps were drawn with R-3.2 language software, GraphPad Prism8, and Visor, respectively.

## Supplementary Information

**Additional file 1: Table S1.** Significantly different primary metabolites between S. salsa and *P. tenuiflora*. **Table S2.** Significantly different phenolic compounds between S. salsa and *P. tenuiflora*.

## Data Availability

All data generated or analyzed during this study are included in this published article and its supplementary information files.

## Abbreviations

*S. salsa*: *Suaeda salsa*
*P. tenuiflora*: *Puccinellia tenuiflora*
OPLS-DA: Discriminant analysis by orthogonal partial least square
SSC: soil alkalization classification standard
