# Supplementary material for: Metabolic differences of two constructive species in saline-alkali grassland in China
Source: BMC Plant Biol. 2022 Jan 26;22:53. doi: 10.1186/s12870-021-03401-y (PMC8790901; doi:10.1186/s12870-021-03401-y)
Supplement: Supplementary file 1 — Additional file 1: Table S1. Significantly different primary metabolites between S. salsa and P. tenuiflora. Table S2. Significantly different phenolic compounds between S. salsa and P. tenuiflora. [file 12870_2021_3401_MOESM1_ESM.docx]

Table S1. Significantly different primary metabolites between *S. salsa* and *P. tenuiflora.*

|  | Metabolite | VIP | Change | *p*-value |
| --- | --- | --- | --- | --- |
| Sugars | sucrose | 1.55 | P>S | *** |
|  | tagatose | 1.48 | P>S | *** |
|  | melezitose | 1.38 | P>S | *** |
|  | D-talose | 1.4 | P>S | ** |
|  | fructose | 1.18 | P>S | *** |
|  | maltotriitol | 1.21 | P>S | ** |
|  | D-galactose | 1.22 | P>S | ** |
|  | sorbose | 1.26 | S>P | *** |
|  | fucose | 1.18 | S>P | ** |
| Amino acids | L-glutamic acid | 1.17 | S>P | *** |
|  | DL-alanine | 1.1 | S>P | *** |
|  | aspartatic acid | 1.2 | P>S | ** |
|  | tyrosine | 1.44 | S>P | ** |
|  | valine | 1.37 | S>P | ** |
|  | homoserine | 1.21 | S>P | ** |
|  | ornithine | 1.2 | S>P | ** |
|  | glycine | 1.24 | S>P | *** |
|  | proline | 1.23 | S>P | ** |
|  | glutamine | 1.21 | S>P | ** |
|  | aminobutyric acid | 1.51 | S>P | ** |
|  | isoleucine | 1.28 | P>S | *** |
|  | norleucine | 1.27 | P>S | ** |
| Alcohols | aminoethanethiol | 1.28 | S>P | ** |
|  | dihydrocarveol | 1.23 | S>P | * |
|  | dodecanol | 1.15 | S>P | ** |
|  | phenylethanol | 1.48 | S>P | *** |
|  | taxifolin | 1.02 | S>P | * |
|  | phytol | 1.09 | S>P | * |
|  | xylitol | 1.17 | P>S | ** |
|  | cuminic alcohol | 1.41 | P>S | ** |
| Acids | gallic acid | 1.34 | S>P | ** |
|  | protocatechuic acid | 1.05 | S>P | *** |
|  | catechol | 1.2 | S>P | *** |
|  | epigallocatechin | 1.11 | S>P | ** |
|  | vanillic acid | 1.16 | S>P | *** |
|  | vinylphenol | 1.38 | S>P | ** |
|  | guaiacol | 1.28 | S>P | ** |
|  | citraconic acid | 1.44 | S>P | * |
|  | malonic acid | 1.05 | S>P | *** |
|  | succinic acid | 1.1 | S>P | ** |
|  | tartaric acid | 1.08 | S>P | ** |
|  | itaconic acid | 1.25 | S>P | * |
|  | pelargonic acid | 1.04 | S>P | ** |
|  | glycolic acid | 1.34 | S>P | * |
|  | 3-hydroxy-3-methylglutaric acid | 1.02 | S>P | ** |
|  | aminooxyacetic acid | 1.32 | S>P | * |
|  | galactonic acid | 1.09 | S>P | *** |
|  | oxalic acid | 1.21 | S>P | ** |
|  | L-gulonic acid | 1.43 | P>S | * |
|  | cumic acid | 1.2 | S>P | ** |
|  | palmitic acid | 1.24 | S>P | * |
|  | methylfumarate | 1.44 | S>P | ** |
|  | gluconic lactone | 1.36 | S>P | * |
|  | methyl hexadecanoate | 1.24 | S>P | * |
|  | dioctyl phthalate | 1.23 | S>P | * |
|  | methyl heptadecanoate | 1.3 | S>P | ** |
|  | nonanoic acid methyl ester | 1.2 | S>P | ** |
|  | methyl octanoate | 1.06 | S>P | ** |
|  | phenylacetic acid | 1.18 | S>P | *** |
|  | hydroxymandelic acid | 1.13 | S>P | * |
|  | 5-hydroxyindole-2-carboxylic acid | 1.25 | S>P | * |
|  | 5-hydroxyindole-3-acetic acid | 1.21 | S>P | * |
|  | 4-hydroxybutyrate | 1.05 | P>S | ** |
|  | L-gulonolactone | 1.07 | P>S | * |
| Others | adipamide | 1.34 | S>P | ** |
|  | tetracosane | 1.05 | S>P | * |
|  | hydroxylamine | 1.21 | S>P | * |
|  | hexadecane | 1.15 | S>P | * |

VIP, variable importance in the projection. *, *p*<0.05; **, *p*<0.01; ***, *p*<0.001.

Table S2. Significantly different phenolic compounds between *S. salsa* and *P. tenuiflora.*

|  | Metabolite | VIP | Change | *p*-value |
| --- | --- | --- | --- | --- |
| C6C1- | protocatechuic acids | 1.25 | P>S | **** |
|  | gallic acid | 1.03 | P>S | ** |
| C6C3- | chlorogenic acid | 1.20 | S>P | **** |
|  | p-hydroxycinnamic acid | 1.13 | S>P | **** |
| C6C3C6- | luteolin | 1.17 | S>P | **** |
|  | quercetin | 1.24 | S>P | * |
|  | myricitrin | 1.06 | S>P | *** |
|  | petunidin | 1.04 | S>P | **** |

VIP: variable importance in the projection. *, *p*<0.05; **, *p*<0.01; ***, *p*<0.001; ***, *p*<0.0001.
